# Cell Cycle Regulating Kinase Cdk4 as a Potential Target for Tumor Cell Treatment and Tumor Imaging

**DOI:** 10.1155/2009/106378

**Published:** 2009-06-17

**Authors:** Franziska Graf, Lena Koehler, Torsten Kniess, Frank Wuest, Birgit Mosch, Jens Pietzsch

**Affiliations:** ^1^Research Center Dresden-Rossendorf, Institute of Radiopharmacy, P.O. Box 510119, 01314 Dresden, Germany; ^2^Department of Oncologic Imaging, Cross Cancer Institute, University of Alberta, 11560 University Avenue, Edmonton, AB, Canada T6G 1Z2

## Abstract

The cyclin-dependent kinase (Cdk)-cyclin D/retinoblastoma (pRb)/E2F cascade, which controls the G1/S transition of cell cycle, has been found to be altered in many neoplasias. Inhibition of this pathway by using, for example, selective Cdk4 inhibitors has been suggested to be a promising approach for cancer therapy. We hypothesized that appropriately radiolabeled Cdk4 inhibitors are suitable probes for tumor imaging and may be helpful studying cell proliferation processes in vivo by positron emission tomography. Herein, we report the synthesis and biological, biochemical, and radiopharmacological characterizations of two ^124^I-labeled small molecule Cdk4 inhibitors (8-cyclopentyl-6-iodo-5-methyl-2-(4-piperazin-1-yl-phenylamino)-8H-pyrido[2,3-*d*]-pyrimidin-7-one (CKIA) and 8-cyclopentyl-6-iodo-5-methyl-2-(5-(piperazin-1-yl)-pyridin-2-yl-amino)-8H-pyrido[2,3-*d*]pyrimidin-7-one (CKIB)). Our data demonstrate a defined and specific inhibition of tumor cell proliferation through CKIA and CKIB by inhibition of the Cdk4/pRb/E2F pathway emphasizing potential therapeutic benefit of CKIA and CKIB. Furthermore, radiopharmacological properties of [^124^I]CKIA and [^124^I]CKIB observed in human tumor cells are promising prerequisites for in vivo biodistribution and imaging studies.

## 1. Introduction

The lack of growth control in tumor cells is a result of alterations in a variety of molecules and regulatory pathways involved in cell cycle control [1]. In particular, the transition from G1 to S phase is deregulated in cancer [2]. Progression of G1 phase is promoted by active complexes of cyclin-dependent kinases (Cdks) and cyclins [2]. Among them serine-threonine kinase Cdk4 triggers an important cascade of events in G1 phase. Cdk4 associated with its cofactor cyclin D catalyzes phosphorylation of retinoblastoma family proteins (pRb). Nuclear proteins pRb exhibit numerous threonine or serine residues as phosphorylation sites [3, 4]. pRb interact as pocket proteins by binding and inhibiting critical regulatory proteins, including transcription factors of E2F family [2]. Thus, pRb act as suppressors of cellular proliferation and regulators of cell cycle in mammalian cells [5]. Phosphorylation of pRb by Cdk4-cyclin D at specific site Ser780 inactivates pRb and leads to release of E2F proteins from the inhibitory pRb-E2F-complex [6]. As a consequence transcription of E2F-regulated genes is promoted. E2F family proteins regulate expression of genes, whose protein products are necessary for cell cycle progression, apoptosis, and DNA repair [7]. Thereby, among other cell cycle dependent gene expressions of E2F-1 or proliferating cell nuclear antigen (PCNA), a cofactor of DNA polymerases that encircles DNA, is activated [8, 9]. 

The disruption and hyperactivation of the Cdk4-cyclin D/pRb/E2F pathway in most cancer types such as malignant sarcomas, gliomas, and breast carcinomas [1, 10–12] have made the Cdk4-cyclin D complex an attractive molecular target for cancer therapy [13]. Specific inhibition of Cdk4-cyclin D results in pRb hypophosphorylation which prevents cell proliferation and further tumor growth. 

In recent years, a large number of selective Cdk4 inhibitors have been described in medicinal chemistry literature [14–16]. In this regard, VanderWel et al. [17, 18] identified various pyrido-[2,3-*d*]pyrimidin-7-ones derivatives as selective inhibitors of the Cdk4-cyclin D complex (IC_50_ 2–32 nM). One of these derivatives (PD 0332991) has entered clinical trials for cancer therapy [15], for example, an accomplished clinical phase I study of oral PD 0332991 in patients with advanced solid tumors, excluding small cell lung cancer and retinoblastoma, and an ongoing study of PD 0332991 in clinical phases I and II in combination with bortezomib and dexamethasone in patients with multiple myeloma. Among this series of compounds, an iodine-containing analog (compound 30 [17]) has also been reported as a potent Cdk4-cyclin D inhibitor (IC_50_ 5 nM) causing inhibition of tumor cell proliferation. The iodine substituent represents an attractive site for an isotopic substitution with several radioiodine isotopes yielding radiolabeled compounds for molecular imaging purposes.

Within the arsenal of molecular imaging methodologies, positron emission tomography (PET) represents an important, noninvasive technique for the development and evaluation of anticancer strategies through visualization and assessment of tissue function and quantification of metabolic pathways in vivo [19]. PET uses short-lived positron emitters like carbon-11 (*t*_1/2_ = 20.39 minutes), fluorine-18 (*t*_1/2_ = 109.77 minutes) and iodine-124 (*t*_1/2_ = 4.18 days) for labeling of biomolecules. The resulting PET radiotracers can be used for in vivo imaging to provide quantitative kinetic information of physiological processes. At present PET represents the most selective and sensitive method (at the picomolar to nanomolar level) to depict physiologic, metabolic, and molecular pathways in vivo. While it lacks the spatial resolution of other imaging techniques like magnetic resonance imaging (MRI) or computed tomography (CT), it is unrivalled in specificity and kinetic sensitivity. Apart from its capability to provide pathophysiological information on human disease, for example, by identification of primary tumors or by tumor staging, PET is also important for the precise assessment of therapeutic efficacy [19]. PET also has become an important technique for the development and evaluation of novel anticancer strategies. Moreover, PET has also entered the field of modern drug development and evaluation. It provides a unique platform to study pharmacokinetics, pharmacodynamics, and the mode of action of novel drugs in both animal models and humans [20].

Most clinical PET studies use 2-[^18^F]fluoro-2-deoxyglucose ([^18^F]FDG) as the radiotracer of choice for tumor imaging. However, although [^18^F]FDG-PET allows tumor imaging with high sensitivity, its application is sometimes limited by its lack of specificity as documented by the inability to discriminate between malignancies and inflammation. Therefore, alternative PET radiotracers are required for specific tumor imaging and further investigation of tumorigenic processes [21].

We hypothesize that appropriately ^124^I-labeled selective Cdk4 inhibitors represent a novel promising class of PET radiotracers for imaging and functional characterization of tumorigenesis in vivo by PET. 

Currently, an exact and accurate assessment of Cdk4 protein levels and/or activity in tumors or other tissues can only be achieved by laborious analyses ex vivo. Moreover, instability of Cdk4 mRNA and protein leads to further difficulties in terms of the intervals between tissue sampling and time of analysis. In this line, noninvasive monitoring by means of nuclear molecular imaging techniques like PET provides unique opportunities to obtain data on functional Cdk4 protein expression or activity during tumorigenesis. Moreover, the development and in vivo study of appropriately radiolabeled selective Cdk4 inhibitors would provide pharmacological data, which may help to understand their exact physiological actions and metabolic pathways.

The aim of our study was the biological, biochemical, and radiopharmacological characterization of two Cdk4 inhibitors (8-cyclopentyl-6-iodo-5-methyl-2-(4-piperazin-1-yl-phenylamino)-8H-pyrido[2,3-*d*]-pyrimidin-7-one (CKIA) and 8-cyclopentyl-6-iodo-5-methyl-2-(5-(piperazin-1-yl)-pyridin-2-yl-amino)-8H-pyrido[2,3-d]pyrimidin-7-one (CKIB)) in different tumor cell lines. 

We investigated cell proliferation, cell cycle distribution, pRb phosphorylation, and mRNA expression of E2F-1 and PCNA in tumor cells after treatment with CKIA and CKIB. Our data demonstrate a definite and specific inhibition of tumor cell proliferation after incubation with CKIA and CKIB. Moreover, radiotracers [^124^I]CKIA and [^124^I]CKIB were used to study uptake in human tumor cells and to investigate in vitro stability. The results are important prerequisites for the design of in vivo biodistribution and imaging studies to further support our hypothesis that radiolabeled Cdk4 inhibitors are suitable radiotracers for tumor imaging by means of PET. 

## 2. Materials and Methods

### 2.1. Radiotracer Synthesis

**Scheme 1:**
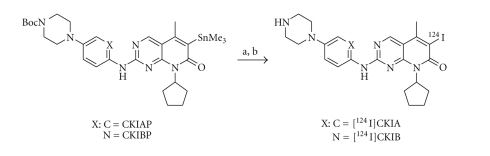
Radiolabeling of CKIAP and CKIBP. a: [^124^I]NaI, Iodogen, rt, 10 minutes; b: TFA, 50°C, 30 minutes.

Compounds [^124^I]CKIA and [^124^I]CKIB were prepared following standard radioiododestannylation protocols as reported by Eisenhut and Mier [22]. Compounds CKIA and CKIB and the corresponding stannylated labeling precursors (CKIAP and CKIBP) were prepared with small modifications according to the procedure described by Barvian et al. [23], and VanderWel et al. [17]. Radiosynthesis of [^124^I]CKIA and [^124^I]CKIB were performed by ^124^I-iodination of the corresponding trimethyl stannylated precursors by regioselective destannylation under mild conditions using [^124^I]NaI and chloramine-T or Iodogen as oxidizing agents (Scheme 1). 

The preparative synthesis starting from 50 *μ*L of CKIAP (5 mg/mL) and 36.08 MBq [^124^I]NaI using Iodogen precoated tubes as oxidizing agent afforded 11.8 MBq (33.6%, decay-corrected) of [^124^I]CKIA within 104 minutes, including HPLC purification. 

The optimized reaction conditions for the preparative [^124^I]CKIA-synthesis were adopted for the synthesis of [^124^I]CKIB. Starting from 50 *μ*L of a 5 *μ*g/*μ*L CKIBP solution in DMSO and 5% glacial acetic acid in methanol (1/3) and 28.89 MBq of [^124^I]NaI within 107 minutes 7.78 MBq (27.8%, decay-corrected) of [^124^I]CKIB was obtained, including HPLC purification. The specific activity was 20 GBq/*μ*mol for [^124^I]CKIA and [^124^I]CKIB, respectively.

### 2.2. Cell Culture

Human tumor cell lines HT-29, a colorectal adenocarcinoma cell line, FaDu, a head and neck squamous cell carcinoma cell line and THP-1, an acute monocytic leukemia cell line, were obtained from DSMZ, Braunschweig, Germany, and cultured in McCoy's 5A medium (HT-29) or RPMI 1640 medium (FaDu, THP-1) supplemented with 10% fetal bovine serum (FBS) and 1% penicillin-streptomycin. THP-1 cells were differentiated with 64 nM 12-O-tetradecanoylphorbol-13-acetate (TPA; Sigma-Aldrich, München, Germany) for 72 hours to macrophage-like cells (THP-1 macrophage). After that, medium containing TPA was removed, and cells were replenished with fresh RPMI medium lacking TPA for respective incubation with CKIA and CKIB (72 hours maximum). During this time THP-1 macrophage cells remain differentiated. All cells were cultured at 37°C, 5% CO_2_, and 95% humidity in a CO_2_ incubator (Heracell, Heraeus, Hanau, Germany).

As a control to classify the effect of CKIA or CKIB, adherent tumor cells were arrested in G1 phase through serum-deprivation. First cells were cultured until confluence and then cell culture medium with only 0.25% FBS was applied for 72 hours.

### 2.3. Cell Growth Studies

Cell growth inhibition was analyzed for up to 72 hours after treatment of CKIA and CKIB. 2.5 × 10^5^ cells were seeded into a cell culture flask and incubated for 24 hours. Then 0.1 or 1.0 *μ*M of the respective compound was added into the cell culture media (either every 24 hours or only once) and cells were counted with the Casy Model TT cell counter (Schaerfe-System, Reutlingen, Germany) after 24, 48, and 72 hours of incubation with CKIA or CKIB.

### 2.4. Flow Cytometry

Cell cycle distribution of the cells was determined by flow cytometry DNA analysis. After treatment of 1 × 10^6^ cells for 24 hours without or with CKIA or CKIB, cells were washed with PBS and detached from the cell culture flask by addition of trypsin (HT-29, FaDu) or accutase (differentiated THP-1). After centrifugation for 5 minutes at 1000 rpm at 4°C cells were washed twice with ice-cold PBS and fixed in 70% ethanol at 4°C for at least 1 hour. Ethanol-fixed cells were washed again, treated with 1 *μ*g/mL ribonuclease I (Sigma-Aldrich) for 30 minutes at 37°C and stained with 10 *μ*g/mL propidium iodide (Sigma-Aldrich) in the dark. Flow cytometry analysis was performed on an FACSCalibur Flow Cytometer (Becton Dickinson, Zürich, Switzerland) by use of the CellQuest Pro software. For cell cycle analysis, 20000 events had been collected in the single-events region with a total event rate not exceeding 300 events/second. Data were analyzed with ModFit LT software package.

### 2.5. SDS-PAGE and Western Blot

At the end of treatment with CKIA or CKIB, cells were washed with ice-cold PBS and lysed in lysis buffer (50 mM Tris-HCl pH 7.5, 150 mM NaCl, 0.5% Nonidet P40 with 1 mM sodium orthovanadate, 1 mM NaF, 10 mM *β*-glycerophosphate, 1 mM dithiothreitol (DTT), 1 mM phenyl-methyl sulfonyl fluoride, 1 mM Pefablock, and 1 *μ*g/mL Leupeptine). Cells were centrifuged at 15000 × g for 15 minutes at 4°C. Protein concentration in the supernatants were quantified using the BCA protein assay kit (Pierce, Rockford, USA) according to the manufacturer's recommendations and bovine serum albumin as protein standard. 

25–50 *μ*g of protein were mixed with sample buffer, denatured at 95°C for 5 minutes and separated on 7.5% sodium-dodecylsulfate (SDS)-polyacrylamide gels for pRb detection. After electrophoresis proteins were transferred to nitrocellulose membranes by electroblotting and blocked for 1 hour at room temperature in TBS/Tween (50 mM Tris-HCl pH 8.0, 150 mM NaCl, 0.05% Tween-20) containing 5% nonfat milk. Membranes were incubated with the primary antibodies Phospho-Rb(Ser780) (1 : 1000, no. 9307 Cell Signaling Technology) or Rb(4H1)mAb (1 : 2000, no. 9309, Cell Signaling Technology) at 4°C over night followed by incubation with secondary peroxidase-conjugated antibodies antirabbit IgG (1 : 10000, A0545, Sigma-Aldrich) or antimouse IgG (1 : 10000, A9044, Sigma-Aldrich) for 1 hour at room temperature. After washing with TBS/Tween proteins were visualized by chemiluminescence using the SuperSignal West Pico Chemiluminescent Substrate (Pierce, Rockford, USA) and exposed to Kodak BioMax light films (Sigma-Aldrich) for 5–15 minutes. In order to detect a second protein, blots were stripped by incubation with 62.5 mM Tris-HCl pH 6.7, 2% SDS and 0.7% *β*-mercaptoethanol at 55°C for 15 minutes. After that, again incubation with primary polyclonal antibody anti-*β*-actin IgG (1 : 1000, A5060, Sigma-Aldrich) and secondary peroxidase-conjugated antibody antirabbit IgG (1 : 10, 000, A0545, Sigma-Aldrich), and protein detection was performed as described above.

### 2.6. RNA Preparation and One-Step Quantitative Real-Time RT-PCR

After 24-hour-incubation with CKIA or CKIB RNA was extracted using the miRNeasy Mini kit (Qiagen, Hilden, Germany) according to the manufacturer's instructions. RNA was treated with RNase free DNase (Fermentas, St. Leon-Roth, Germany) according to the manufacturer's guidelines to prevent genomic DNA contamination. The reverse transcription and quantitative real-time PCR of specific mRNA was carried out in one step from 50 ng of total RNA and intron-spanning primers (Metabion, Munich, Germany) using the QuantiTect SYBR Green RT-PCR kit (Qiagen) according to the manufacturer's instructions. The primer sequences and annealing temperatures are given in Table 1. One-step quantitative real-time RT-PCR was performed in a mastercycler ep Realplex system (Eppendorf, Hamburg, Germany). RNA samples were reverse transcripted for 20 minutes at 50°C. Then, quantitative real-time PCR was performed for 40 cycles with denaturation at 95°C for 20 seconds, annealing at 56 to 59°C (depending on the primers, Table 1) for 20 seconds and extending at 68°C for 30 seconds. All PCR products were checked by melting point analysis upon completion of PCR cycles by heating up from 55 to 95°C in 20 minutes. 

For each cell line and treatment two to four different RNA samples were analyzed as triplicates. A negative control without RNA template was included in every measurement for the different primers. For each experimental sample, mRNA levels were normalized to *β*-actin mRNA levels (threshold cycle *β*-actin—threshold cycle mRNA of interest = Δ*C*_*t*_). Therefore primers for the amplification of *β*-actin were included in every measurement to correct for sample-to-sample variations. mRNA levels were presented as 2^ΔΔ*C*_*t*_^ (ΔΔC_*t*_ = Δ*C*_*t*_ (treatment) − (Δ*C*_*t*_(control)).

### 2.7. Stability and Radiotracer Uptake Studies

 Stability of [^124^I]CKIA and [^124^I]CKIB was examined after 1 hour at 37°C in different physiological buffers (pH 4.2, 7.4, 9.0), cell culture medium, and rodent plasma by HPLC analysis. 

For radiotracer uptake experiments, 5 × 10^4^ cells were seeded in a cavity of a 24-well plate (Greiner, Frickenhausen, Germany) and cultured in a humidified atmosphere of 5% CO_2_ at 37°C using the above-mentioned cell culture media. After overnight incubation, 0.5 mL of cell culture medium with about 30 kBq [^124^I]CKIA or [^124^I]CKIB was added and incubation was continued for several time points at 37°C and 4°C. Subsequently, cells were washed three times with ice-cold PBS and lysed in 0.5 mL 0.1 M sodium hydroxide with 1% SDS. Cell lysates were counted with a Cobra II gamma counter (Canberra-Packard, Meriden, CT, USA). Protein levels were quantified using the BCA protein assay kit (Pierce, Rockford, USA) according to the manufacturer's recommendations and bovine serum albumin as protein standard. Uptake data for all experiments are expressed as percent of injected dose per mg protein (%ID/mg protein).

### 2.8. Statistical Analysis

Descriptive data were expressed as arithmetic means ± standard deviations. The number of *n* in the figure legends represents the number of independent experiments. Statistical analyses were performed using one-way ANOVA or Wilcoxon signed rank test. ANOVA was coupled with a post hoc Bonferroni correction analysis when appropriate. Spearman's rank correlation coefficient was calculated between data related to Cdk4 activity and radiotracer uptake. For all analyses a value of *P* < .05 was considered as statistically significant. The SPSS statistical software package (version 12.0 for Windows; SPSS Inc., Chicago, IL, USA) was used for all analyses.

## 3. Results

### 3.1. Cell Growth Studies

For the characterization of cell cycle inhibitors CKIA and CKIB three different tumor cell lines were studied with respect to their concentration dependent cell proliferation. Cell growth studies showed very different growth curves for all tumor cell lines. HT-29 cells show with 17 hours the shortest doubling time. For FaDu cells a doubling time of 22 hours and for THP-1 cells of 38 hours could be observed in logarithmic phase. For all experiments with CKIA and CKIB tumor cells were in logarithmic growth phase. After 72 hours of incubation with TPA, THP-1 monocyte-like suspension cells adhere almost completely at the bottom of culture flask and differentiate to macrophage-like cells. TPA differentiated THP-1 cells (THP-1 macrophage) showed no more cell proliferation and served as control cell model. 

**Figure 1:**
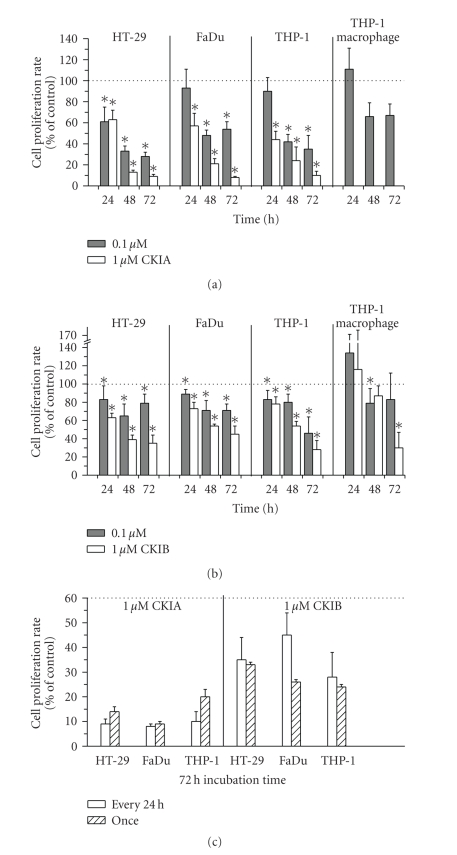
Cell proliferation rate of HT-29, FaDu, THP-1, and THP-1 macrophage after treatment with (a) CKIA or (b) CKIB (every 24 hours) in percentage of control without treatment. (c) Comparison of daily treatment and single treatment after 72 hours of incubation with 1 *μ*M CKIA or CKIB, respectively. (means ± standard deviations, *n* ≥ 6, ANOVA, *P < .05, compared to control (100%)).

Tumor cell growth studies with CKIA (every 24 hours treatment) indicated a significantly reduced cell proliferation in all tumor cell lines at 48 and 72 hours after treatment with 0.1 (<50%) and 1 *μ*M (<25%) of CKIA (Figure 1, *P* < .05). Nonrecurring treatment with 1 *μ*M of CKIA for 72 hours provided similar findings compared to treatment every 24 hours. Also in THP-1 macrophages, cell number was decreased by 33% with 0.1 *μ*M of CKIA (Figure 1).

Generally, CKIB showed similar effects on cell proliferation, albeit these effects were only achieved at higher CKIB concentrations or by longer incubation time compared to CKIA. After 48 hours of treatment with CKIB, cell proliferation in HT-29 cells was reduced by 35% (0.1 *μ*M) and 61% (1.0 *μ*M), respectively, in FaDu cells 29% (0.1 *μ*M) and 46% (1.0 *μ*M), respectively, and in THP-1 cells 20% (0.1 *μ*M) and 46% (1.0 *μ*M), respectively (Figure 1, *P* < .05). 72 hours after treatment with 1.0 *μ*M of CKIB cell proliferation was decreased to 35, 45, or 28% (HT-29, FaDu, THP-1). Cell number of THP-1 macrophage cells for this incubation condition was also significantly depleted. 

### 3.2. Cell Cycle Analysis

**Figure 2:**
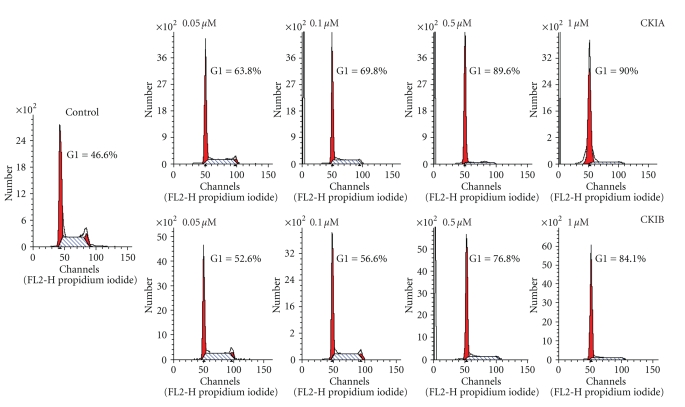
Cell cycle distribution of HT-29 cells at 24 hours after treatment with CKIA or CKIB (representative histograms).

CKIA and CKIB were analyzed for their effects on cell cycle distribution. Already 24 hours after incubation with CKIA the percentage of tumor cells in G1 phase showed a concentration dependent increment of up to 89.9% in HT-29 and 84.5% in FaDu tumor cells (Figure 2, Table 2, *P* < .05). 

CKIA induced a G1 phase arrest after incubation with 0.05, 0.1, and 0.5 *μ*M in THP-1 cells. At higher concentration of 1.0 *μ*M, however, cell cycle distribution switched back to values obtained for untreated cells. Over 89% of HT-29 and THP-1 cells in G1 phase were obtained after 24 hours of incubation with 1.0 *μ*M of CKIB (Table 2, *P* < .05). A concentration dependent alteration of cell cycle distribution after incubation with CKIB was also observed in FaDu cells, but 24 hours after incubation with 1.0 *μ*M of CKIB only 70.7% were achieved in G1 phase. THP-1 macrophage cells showed no change in percentage of G1 phase neither after incubation with CKIA nor with CKIB.

### 3.3. pRb Phosphorylation

**Figure 3:**
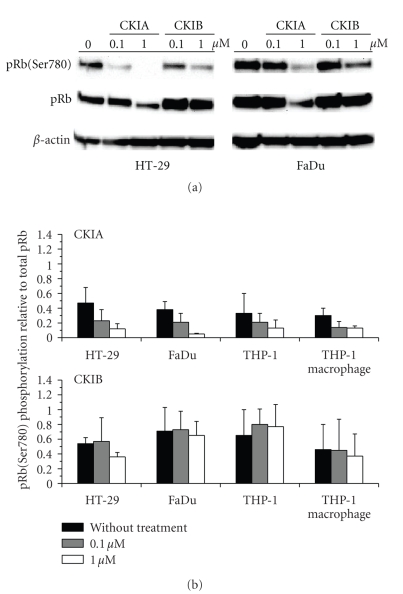
(a) Representative Western blots of HT-29 and FaDu cell lysates. (b) pRb phosphorylation at Ser780 relative to total pRb (means ± standard deviations, *n* = 2–5).

To study the interaction of CKIA or CKIB with Cdk4 and the influence on the downstream signaling pathway, detection of pRb phosphorylation status in whole cell lysates was performed by Western Blot. Cdk4 specific phosphorylation of pRb on Ser780 and were normalized to total amount of pRb, *β*-actin served as internal control. In all cell lines antibodies Phospho-Rb(Ser780) and Rb(4H1)mAb only recognized endogenous pRb species with a size of 110 kDa. The total levels of pRb remained constant in all cell lines, only incubation with 1 *μ*M CKIA by trend showed a decrease of total pRb. After incubation with CKIA a concentration dependent hypophosphorylation of pRb(Ser780) was found (Figure 3). pRb(Ser780) phosphorylation was decreased three- to tenfold after 24 hours of treatment with 0.1 and 1 *μ*M CKIA in all cell lines. Results of pRb(Ser780) phosphorylation analyses suggested a downregulation after 24 hours of incubation with 1 *μ*M of CKIB in HT-29 cells (Figure 3). No significant change in pRb(Ser780) phosphorylation status of FaDu and THP-1 cells incubated with CKIB was found.

### 3.4. E2F-1 and PCNA mRNA Expression

**Figure 4:**
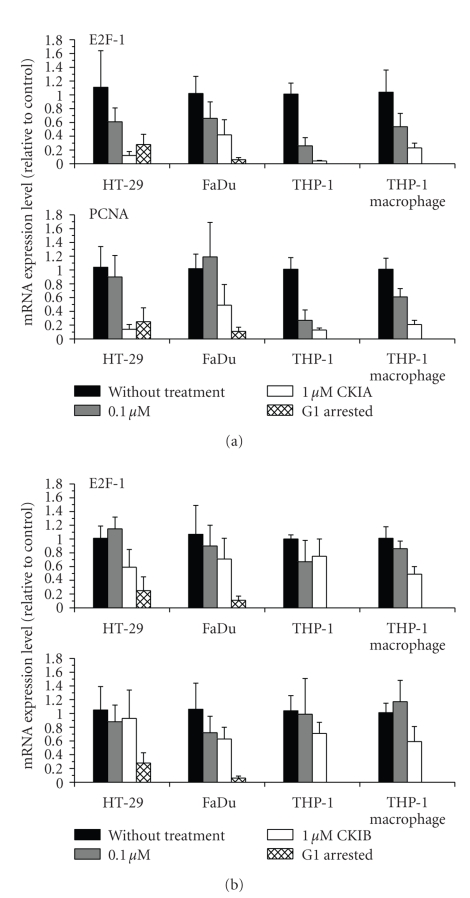
mRNA expression level of E2F-1 and PCNA after 24-hour-incubation with (a) CKIA or (b) CKIB and in G1 arrested adherent tumor cells after serum-deprivation relative to control cells without treatment (means ± standard deviations, *n* ≥ 6).

mRNA expression of pRb effected E2F-1 and PCNA genes was measured in relation to the effects of CKIA and CKIB on Cdk4 downstream signaling pathway. For grading and comparison of the studied effects also mRNA levels of serum-deprived G1 arrested adherent cells were detected. A substantial downregulation of E2F-1 and PCNA mRNA expression could be demonstrated after incubation with 1 *μ*M of CKIA in all cell lines studied (Figure 4). 

In HT-29 and THP-1 tumor cells an up to 15% reduction of mRNA expression for both genes (E2F-1 and PCNA) compared to control without treatment was detectable. In HT-29 and FaDu cells mRNA expression of E2F-1 and PCNA was in the range of corresponding levels in G1 arrested cells. Further analysis of released serum-deprived HT-29 cells after 24 hours incubation with CKIA exhibited a consistent downregulation of both E2F-1 and PCNA mRNA (data not shown). Also E2F-1 and PCNA mRNA expression analyses after 24 hours of incubation with 1 *μ*M of CKIB indicated a downregulation in all cell lines and THP-1 macrophages. Expression of E2F-1 and PCNA mRNA was decreased by CKIB up to 60% in FaDu or HT-29 cells, but without statistical significance.

### 3.5. Stability and Radiotracer Uptake Studies

Preliminary experiments with [^124^I]CKIA and [^124^I]CKIB indicated a sufficient stability of this compounds in various buffers (pH 4.2, 7.4, and 9.0), cell culture media, and rodent plasma samples in the range of 93–97% at 37°C for 1 hour at minimum.

**Figure 5:**
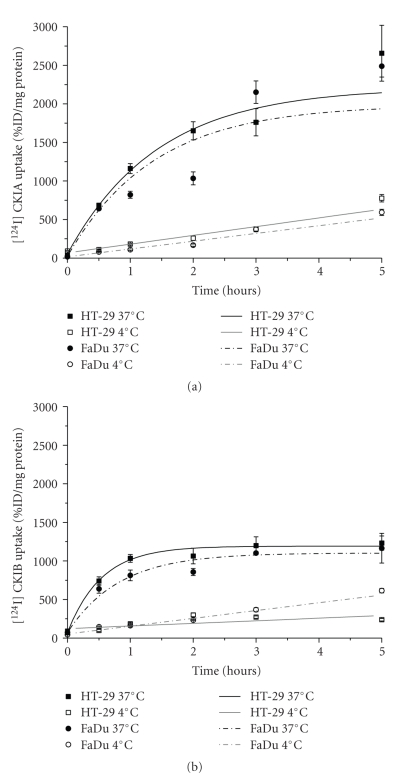
Cellular radiotracer uptake of (a) [^124^I]CKIA and (b) [^124^I]CKIB over a period of 5 hours at 37°C and 4°C (means ± standard deviations, *n* = 4, ANOVA, *P* < .05, 4°C compared to 37°C, resp.). Symbols represent observed data, lines represent computer-derived fits.

In vitro radiotracer uptake studies in adherent tumor cells using [^124^I]CKIA and [^124^I]CKIB showed substantial uptake in HT-29 and FaDu cells (Figure 5). 

The time-dependent cellular uptake was similar in both cell lines and uptake of [^124^I]CKIA is steadily increased with time up to 5 hours. After 2 hours at 37°C 1649 ± 117 %ID/mg protein in HT-29 and 1033 ± 84 %ID/mg protein in FaDu cells were obtained. At 4°C an obvious lower uptake was detectable in both cell lines (258 ± 30 %ID/mg protein in HT-29, 169 ± 14 %ID/mg protein in FaDu, *P* < .05). In vitro studies with [^124^I]CKIB demonstrated a radiotracer uptake of 904 ± 43 %ID/mg protein in HT-29 and 856 ± 45 %ID/mg protein in FaDu cells after 2 hours at 37°C. Radiotracer uptake of [^124^I]CKIB was only marginally increased up to 5 hours at 37°C and again radiotracer uptake at 4°C was substantially lower compared to 37°C (*P* < .05).

## 4. Discussion

In 2005, CKIA was reported as a specific and potent Cdk4 inhibitor with an IC_50_ value of 5 nM by VanderWel et al. [17], but in spite of promising properties only marginal examinations concerning inhibition of tumor cell proliferation were performed. 

The purpose of our study was the further evaluation of the known Cdk4 inhibitor CKIA and of a new derivative compound CKIB concerning their biological, biochemical, and radiopharmacological properties, to proof the feasibility and suitability of these Cdk4 inhibitors for radiotracer development and imaging of cell proliferation processes in vivo especially in tumor entities. CKIB was designed as a less lipophilic analog of Cdk4 inhibitor CKIA to reduce nonspecific binding. 

To the best of our knowledge, for the first time Cdk4 inhibitors CKIA and CKIB were demonstrated to act as tumor cell growth inhibitors via specific inhibition of Cdk4/pRb/E2F signaling pathway and induction of G1 arrest in cells.

For our studies, HT-29, FaDu, and THP-1 cells were used as continuously proliferating cell models. The three tumor cell lines represent common models for human solid tumors (HT-29, FaDu) and leukemia (THP-1). Furthermore, TPA differentiated THP-1 cells served as a cellular model that shows no cell proliferation potential due to their arrest in cell cycle. The tumor cell lines studied exhibited different doubling rates, which could influence the impact of cell cycle inhibitors. However, cell proliferation after treatment with CKIA or CKIB showed no conspicuous differences between rapid proliferating HT-29 or slower proliferating FaDu and THP-1 cells, respectively. In all cell lines a significant concentration and time dependence of cell division was observed after application with CKIA or CKIB. Indeed, CKIB is not as potent as CKIA to inhibit cell growth. After 72 hours of incubation with 1.0 *μ*M of CKIA or CKIB a decrease in cell number about 55–90% compared to control without treatment could be found in tumor cells. CKIA and CKIB inhibit cell division, but no decrease of cell number under the seeded cell number was observed after incubation with CKIA or CKIB. Control cells THP-1 macrophage only show decreased cell numbers after 72 hours of incubation with CKIB. Hence, no acute cytotoxic effect of CKIA or CKIB is assumed. Interestingly, also nonrecurring treatment with 1.0 *μ*M of compounds for 72 hours showed similar cell number values as for ever 24 hours treatment, which could be an indication for good availability and/or stability of active CKIA and CKIB in the cells. It is likely, because of action of CKIA and CKIB in the nucleus, that these compounds are not accessible for transport outside of cells, for example, by multidrug resistance proteins (MDR). 

Measurement of cell cycle distribution clarified and complemented the observed data of cell growth studies. 24 hours after incubation with CKIA or CKIB a concentration dependent increment of cells in G1 phase was detectable in all tumor cell lines. Cell cycle distribution of THP-1 macrophage cells remained constantly. Unlike to cell growth studies, differences between the tumor cell lines after treatment with a certain concentration of CKIA or CKIB were found. Cell cycle distribution studies demonstrated a lower increment of percentage of FaDu cells in G1 phase compared to HT-29 or THP-1 cells. For example, 24 hours after incubation with 0.1 *μ*M CKIA percentage of cells in G1 phase raised up 10.5% in FaDu, 22.5% in HT-29, and 33.4% in THP-1 cells. Surprisingly, at a concentration of 1.0 *μ*M of CKIA cell cycle distribution values in THP-1 cells were observed similar to untreated cells. A comparable phenomenon was obtained by Toogood et al. [18], in human breast carcinoma cells MDA-MB453 after treatment with other pyrido[2,3-*d*]pyrimidin-7-one derivatives identified as Cdk4 inhibitors. This unexpected characteristic of cell cycle distribution observed for a few Cdk4 inhibitors at higher concentrations (>1.0 *μ*M) is still not understood and requires further investigations to clarify this phenomenon.

The effect of CKIA on cell cycle distribution was considerably higher compared to CKIB at the same concentration range. Nevertheless, both CKIA and CKIB caused a cell cycle arrest in G1 phase (G1 ≥ 85%) in tumor cell lines HT-29, FaDu, and THP-1, although a higher concentration of CKIB is required to obtain the same extent of cell cycle arrest. Only in FaDu cells CKIB failed to reach an increment of G1 phase exceeding 85% (achieved for 1 *μ*M CKIA) under conditions determined. After incubation with 1.0 *μ*M CKIB only 70.7% of FaDu cells were achieved in G1 phase.

To further characterize the cell cycle arrest, we analyzed the Cdk4 specific pRb phosphorylation status and mRNA levels downstream of Cdk4-cyclin D/pRb pathway. Rb gene is mutated in some types of cancer [24, 25] and nonfunctional pRb evades regulation through Cdk-induced phosphorylation [26]. As inhibitors for the Cdk4-cyclin D/pRb pathway, compounds CKIA and CKIB should inhibit cell proliferation and pRb phosphorylation of cells with functional pRb. HT-29 and FaDu are tumor cell lines with normal pRb status [27, 28], and also in THP-1 cells full length pRb is found, which indicate the existence of functional pRb in these tumor cell line [29]. More than ten phosphorylation sites of pRb are known in vivo [3]. During G1/S transition Cdk4/6-cyclin D and Cdk2-cyclinA/E are involved in pRb phosphorylation, but different specificity of serine residues of pRb were identified for these different Cdk-cyclin-complexes [4, 30]. While Cdk4-cyclin D efficiently and specifically phosphorylates pRb on Ser780 and Ser795, Cdk2-cyclin A/E does not. Analyses of pRb phosphorylation resulted in a significantly decreased Ser780 phosphorylation in all cell lines studied dependent on concentration of CKIA. 

After 24 hours of incubation with CKIB a tendency of pRb hypophosphorylation on Ser780 in HT-29 and THP-1 macrophage cells was observed. Additional experiments with specific antibodies against phospho-Ser795 of pRb also demonstrated a CKIA concentration dependent decrease of pRb phosphorylation. The cell cycle regulatory activity of pRb is especially mediated through its binding of transcriptional factors like E2F, which regulates growth-promoting genes [5]. pRb phosphorylated on Ser780 cannot bind to E2F transcription factors in vivo [30] and as a consequence of pRb release from the pRb-E2F complex mRNA expression of E2F regulated genes, for example, E2F-1 or PCNA is activated [8, 31]. A substantial downregulation of both E2F-1 and PCNA mRNA expression was found after 24 hours of incubation with CKIA in all cell lines. The mRNA levels in HT-29 and FaDu with 1.0 *μ*M of CKIA are comparable to that of serum-deprived cells arrested in G1 phase. Also after treatment with 1.0 *μ*M of CKIB a decreased mRNA expression of E2F-1 and PCNA was detectable. In summary, our data report a distinct inhibition of pRb phosphorylation by Cdk4-cyclin D as the consequence of CKIA and CKIB effects in cells. Reduced pRb phosphorylation leads to a disruption of the E2F cascade, exemplified for E2F-1 and PCNA mRNA expression. It can be assumed that other genes downstream of Cdk4-Cyclin D/pRb/E2F pathway are affected through CKIA and CKIB induced inhibition of pRb phosphorylation, for example, genes essential for DNA duplication and cell cycle control like DNA polymerase *α* and cyclin E. The influence of CKIA and CKIB to the Cdk4-cyclin D/pRb/E2F pathway finally ends in cell cycle arrest and a stop of cell proliferation.

CKIA, a pyrido[2,3-*d*]pyrimidin-7-one derivative, was reported by VanderWel et al. [17], in 2005 for the first time as a specific and potent Cdk4 inhibitor with an IC_50_ value of 5 nM. All derivative compounds synthesized by this group were classified concerning their inhibition of different kinases by means of IC_50_ values measured by in vitro kinase assays. IC_50 _ values allow a comparison between different compounds, but no inferential evidence about their properties in cells can be made. Furthermore, cell effective concentration is much higher than IC_50_ value determined by an in vitro kinase assay, due to the two barriers cell membrane and nuclear membrane that have to be overcome, and possible metabolism of the compound within the cell. Effective concentration also depends on tumor cell type and tissue origin. Hence, IC_50_ values determined by in vitro kinase assays should not be the only discriminating argument for choice of a suitable compound for therapeutic applications. 

In our opinion, flow cytometry is a meaningful, fast, and reproducible method for evaluation of selective cell cycle inhibitors concerning their effect in cells. Previous evaluation of CKIA in consideration of tumor cell growth inhibition of human colon carcinoma cell line HCT116, measuring the [^14^C]-thymidine incorporation, resulted in an IC_50 _value of 0.104 *μ*M [17]. Although a direct comparison is not possible because of variations in methods and conditions, this finding correlates very well with our data observed for human colon carcinoma cell line HT-29 by flow cytometry. In HT-29, an ED_50_ value of 0.095 *μ*M CKIA could be calculated for increased percentage of cells in G1 phase. In conclusion, among others flow cytometry would be a good alternative for inhibitor classification.

However, VanderWel et al. has focused on other derivatives of pyrido[2,3-*d*]pyrimidin-7-one, regarding to the reduced selectivity of CKIA compared to other compounds investigated [17]. PD 0332991 has been extensively characterized among other highly selective inhibitors concerning biochemical and biological properties in vitro and in vivo [15, 32]. In consequence PD 0332001 was scheduled for clinical trials in 2004 in combination with common chemotherapeutics. The question to be answered is, if exceeding high selectivity of pyrido[2,3-*d*]pyrimidin-7-one derivatives against other Cdks than Cdk4 will be of outstanding importance for tumor treatment. An expanded effect on different Cdks could also be imaginable for effective tumor cell growth inhibition.

In summary, CKIA and also the new compound CKIB were demonstrated to act as tumor cell growth inhibitors via specific inhibition of Cdk4-Cyclin D/pRb/E2F signaling pathway. The mechanism of action confirms the therapeutic benefit of these compounds in field of cancer treatment, although their lowered selectivity. 

We hypothesized that potent Cdk4 inhibitors, which are suitable for cancer therapy, are also of interest as radiotracers for imaging of cell proliferation processes in vivo and especially for tumor visualization by positron emission tomography (PET). 

For PET imaging application, beside the knowledge about biological and biochemical effectiveness of potent Cdk4 inhibitors also radiopharmacological characterization of respective radiotracers is required. 

Based on the structure of the two compounds CKIA and CKIB appropriate radiolabeling strategies were designed and radiolabeling with the positron-emitting nuclide iodine-124 was performed (decay-corrected radiochemical yields: 33.6% [^124^I]CKIA, and 27.8% [^124^I]CKIB, purity: >98%, resp.). Using [^124^I]CKIA and [^124^I]CKIB stability and radiotracer uptake studies were performed in HT-29 and FaDu cells. In both tumor cell lines a substantial cellular uptake of [^124^I]CKIA and [^124^I]CKIB was observed at 37°C. The early uptake kinetics of both compounds was comparable in both tumor cell lines. In posterior phase (>1 hour) [^124^I]CKIA showed a higher cellular uptake compared to [^124^I]CKIB. Of note, [^124^I]CKIB uptake level remained nearly constant after 1 hour. This is consistent with the different therapeutic potency of CKIA and CKIB observed in tumor cell inhibition studies. At lower temperature of 4°C obvious lower uptakes were detected for both compounds. This finding indicates an energy dependent mechanism for uptake into cells. In the literature no analysis or information concerning transport mechanism of pyrido[2,3-*d*]pyrimidin-7-one derivatives were published. Pyrido[2,3-*d*]pyrimidin-7-one derivatives were predicted to bind ATP site of Cdks [23], thus implying a structural similarity to ATP. Nucleoside transporters (NTs) mediate the uptake of physiologic nucleosides like adenosine and were also reported to transport anticancer nucleoside drugs [33]. Especially, energy-dependent concentrative nucleoside transporters (CNTs) could be presumed for CKIA and CKIB transport into cells. Since no specific pharmacological inhibitor has been found for CNTs yet [34], it is difficult to confirm this presumption. Another transporter identified to transport nucleotide analogs is MDR5 [35]. Nevertheless, the putative transporters still have to be identified. The identification of specific transporters of pyrido[2,3-*d*]pyrimidin-7-one derivatives will contribute to understanding of effectiveness of these compounds and especially for radiotracer uptake a valuation of tumor accumulation could be made depending on transporter frequency of tumor species.

Correlation analysis only showed a weak correlation between E2F-1 mRNA expression as measure of Cdk4 enzyme activity and CKI (CKIA and CKIB) uptake (*τ* = 0.390, *P* = .042, *n* = 15). According to this, the transport of both radiotracers into the cells would be the rate determining step for cellular uptake of CKIA and CKIB at least in monolayer cell cultures.

The criteria for therapeutic and imaging agents are very different. A therapeutic active compound must reach and affect a certain target, whereas the agent for imaging only has to be retained by the target [36]. Certainly, many other properties of a radiotracer like biodistribution or lipophilicity contribute to a specific tumor accumulation and are relevant for high-contrast images. The inhibitory effect of CKIA or CKIB would only be of subordinate interest regarding the imaging application of these compounds. Nevertheless, high specificity and selectivity are a prerequisite for specific accumulation in tumor cells. However, a less selective or specific compound with better biodistribution properties but comparable tumor uptake could have advantages for the application as radiotracer for imaging of tumors by PET.

In summary, our data demonstrate a definite and specific inhibition of tumor cell proliferation when incubated with CKIA and CKIB due to an arrest of tumor cells in G1 phase. The repression of G1 progression is achieved by inhibition of the Cdk4-cyclin D/pRb/E2F pathway. The tumor cell growth inhibition observed in vitro point out the potential therapeutic benefit of these compounds, which has to be proven in further experiments in vivo. 

The radiotracer uptake observed in human tumor cells and the stability of [^124^I]CKIA and [^124^I]CKIB as found in physiological buffers are promising prerequisites for in vivo biodistribution and imaging studies by PET. An interesting effect arising from the radiotracer uptake studies using [^124^I]CKIA and [^124^I]CKIB is the observed energy dependent transport of CKIA and CKIB, due to the difference of uptake at 37°C and 4°C. The putative transporters still have to be identified. In this regard, and to further support our hypothesis that radiolabeled Cdk4 inhibitors are suitable radiotracers for tumor imaging, comprehensive in vivo experiments of [^124^I]CKIA and [^124^I]CKIB involving biodistribution and small animal PET studies [37] should be performed.

## Figures and Tables

**Table 1:** Primer sequences and annealing temperatures for quantitative real-time RT-PCR.

Primer	bp pos.	*T*_annealing_/°C	Sequence
*β*-actin-f	736 – 755	59	5^*′*^-GGA CTT CGA GCA AGA GAT GG-3^*′*^
*β*-actin-r	969 – 950		5^*′*^-AGC ACT GTG TTG GCG TAC AG-3^*′*^
E2F-1-f	796 – 815	58	5^*′*^-AGC TGG ACC ACC TGA TGA AT-3^*′*^
E2F-1-r	945 – 926		5^*′*^-GAG GGG CTT TGA TCA CCA TA-3^*′*^
PCNA-f	444 – 463	56	5^*′*^-GGC GTG AAC CTC ACC AGT AT-3^*′*^
PCNA-r	688 – 669		5^*′*^-TCT CGG CAT ATA CGT GCA AA-3^*′*^

**Table 2:** Percentage of cells in G1 phase after 24 hours of treatment with different concentrations of CKIA or CKIB (values are means ± standard deviations in %, *n* ≥ 8, *P < .05, compared to control (0 *μ*M), resp.).

CKIA/*μ*M	0	0.05	0.1	0.5	1.0
HT-29	45.6 ± 4.1	62.3 ± 4.0*	68.1 ± 4.1*	90.1 ± 1.4*	89.9 ± 0.8*
FaDu	46.4 ± 4.7	55.7 ± 6.5*	56.9 ± 6.5*	82.2 ± 2.5*	84.5 ± 3.1*
THP-1	58.8 ± 4.5	87.3 ± 8.0*	92.1 ± 3.2*	86.2 ± 5.2*	61.7 ± 6.4
THP-1 macrophage	46.9 ± 5.9	45.1 ± 1.8*	51.9 ± 3.3	45.3 ± 1.5*	41.4 ± 2.0*

CKIB/*μ*M	0	0.05	0.1	0.5	1.0

HT-29	46.7 ± 2.4	54.0 ± 1.5*	58.7 ± 2.7*	81.5 ± 4.9*	89.9 ± 4.3*
FaDu	58.6 ± 3.4	64.7 ± 3.5*	65.8 ± 5.4*	69.4 ± 6.1*	70.7 ± 3.6*
THP-1	59.9 ± 1.8	68.6 ± 2.8*	73.5 ± 1.0*	86.8 ± 1.7*	89.1 ± 1.0*
THP-1 macrophage	52.2 ± 3.2	53.2 ± 2.4	53.6 ± 2.0	55.4 ± 2.0	54.6 ± 2.7

## Abbreviations

Cdk: Cyclin-dependent kinase
E2F: E2 promoter binding factor
MDR: Multidrug resistance protein
PCNA: Proliferating cell nuclear antigen
PET: Positron emission tomography
pRb: Retinoblastoma protein.
